# Aspartame in conjunction with carbohydrate reduces insulin levels during endurance exercise

**DOI:** 10.1186/1550-2783-9-36

**Published:** 2012-08-01

**Authors:** Jason Siegler, Keith Howell, Rebecca Vince, James Bray, Chris Towlson, Daniel Peart, Duane Mellor, Stephen Atkin

**Affiliations:** 1 School of Science and Health, University of Western Sydney, Campbelltown, Australia; 2 Diabetes and Endocrinology, Hull York Medical School, University of York, York, United Kingdom; 3 Department of Sport, Health and Exercise Science, University of Hull, Hull, United Kingdom; 4 Clinical Sciences Department, University of Chester, Chester, United Kingdom; 5 School of Science and Health, University of Western Sydney, Campbelltown Campus, Locked Bag 1797, Penrith, NSW, 2751, Australia

**Keywords:** Aspartame, Exercise, Insulin, Blood glucose

## Abstract

**Background:**

As most sport drinks contain some form of non-nutritive sweetener (e.g. aspartame), and with the variation in blood glucose regulation and insulin secretion reportedly associated with aspartame, a further understanding of the effects on insulin and blood glucose regulation during exercise is warranted. Therefore, the aim of this preliminary study was to profile the insulin and blood glucose responses in healthy individuals after aspartame and carbohydrate ingestion during rest and exercise.

**Findings:**

Each participant completed four trials under the same conditions (45 min rest + 60 min self-paced intense exercise) differing only in their fluid intake: 1) carbohydrate (2% maltodextrin and 5% sucrose (C)); 2) 0.04% aspartame with 2% maltodextrin and 5% sucrose (CA)); 3) water (W); and 4) aspartame (0.04% aspartame with 2% maltodextrin (A)). Insulin levels dropped significantly for CA versus C alone (43%) between pre-exercise and 30 min, while W and A insulin levels did not differ between these time points.

**Conclusions:**

Aspartame with carbohydrate significantly lowered insulin levels during exercise versus carbohydrate alone.

## Findings

### Background

The intra-individual variability recently reported with aspartame ingestion, blood glucose regulation and insulin secretion has raised doubts about the appropriateness of this sweetener as a substitute for sucrose in the diet [1]. Ferland and colleagues have reported aspartame to induce similar increases in blood glucose and insulin levels to that of sucrose after a meal in type 2 diabetics [1]. Variation between responses with aspartame consumption is particularly important when considering the impaired glucose tolerance (IGT) in β-cell function and the decreased peripheral insulin resistance that exists in most type 2 diabetics [2].

The addition of regular, physical exercise in conjunction with dietary interventions is often prescribed as a non-pharmaceutical approach to controlling blood glucose in IGT individuals and type 2 diabetics [2]. Exercise has been shown to decrease blood glucose in this population through the upregulation of monocarboxylic transporters (e.g. GLUT 4) to the plasma membrane as well as improved insulin sensitivity [3]. However it is this additional regulatory support through GLUT 4 transporters that may also make some individuals susceptible to hypoglycemia post-exercise if not managed appropriately [4]. In reality, it is common for individuals to consume sport drinks either during and/or after an exercise session. As some sport drinks may contain various forms of non-nutritive sweeteners such as aspartame (e.g. Lucozade Sport®), and with the reported irregularities in blood glucose regulation and insulin secretion associated with aspartame, a further understanding of the effects on insulin and blood glucose regulation during such conditions is warranted. Therefore, the aim of this preliminary study was to profile the insulin and blood glucose responses in healthy individuals after aspartame and carbohydrate ingestion during rest and exercise. We hypothesized that insulin and blood glucose responses would differ between the aspartame and carbohydrate conditions during both rest and exercise.

### Methods

Nine healthy, recreationally active males (age: 22 ± 2 years; height: 180 ± 9 cm; weight: 78.6 ± 8.5 kg; participating in regular physical exercise at least twice per week) volunteered to take part in the study after being informed verbally and in writing as to the nature and risks associated with the study. Participants were free of any cardiac or metabolic diseases, did not smoke, and refrained from supplementation of all kinds (i.e., vitamins, ergogenic aids, etc.) during the testing period. All signed informed consent and the study was approved by the Departmental Human Ethics Committee and followed the principles outlined by the Declaration of Helsinki.

### Experimental protocol

Following a familiarization session (approximately one week) in which all participants cycled the 60 minute exercise requirement, each participant completed four trials in a climate controlled laboratory separated by seven to ten days (balanced Latin squares design) under the same conditions differing only in their fluid intake: 1) carbohydrate (2% maltodextrin and 5% sucrose (C)); 2) 0.04% aspartame with 2% maltodextrin and 5% sucrose (CA)); 3) water (W); and 4) aspartame (0.04% aspartame with 2% maltodextrin (A)). Participants were instructed to follow the same diet and training schedule for the three days prior to each experimental trial.

Each participant reported to the laboratory in the morning after a 12-hour overnight fast, consuming only water in the intervening period. After sitting for ten minutes, a basal (baseline) 5 mL venous blood sample was obtained from an antecubital vein via vaccuette into serum separator tubes for subsequent analysis of serum insulin as well as a capillary sample for blood glucose (BG) (YSI 2300 stat plus glucose-lactate analyzer, YSI inc., Yellowsprings, Ohio, USA). Due to ethical constraints, the total number of venous samples was limited to four (baseline, pre-exercise, 30 minutes and post-exercise). Therefore, we were restricted to only profiling the blood glucose response with capillary sampling during resting (every 10 minutes) and exercise conditions (matched to venous sampling for insulin comparison). The test drinks were then provided in a randomized, double blind order with an initial 7 ml·kg^-1^ of body weight (BW), followed by seated rest for 45 minutes before they commenced the exercise protocol. Capillary blood was sampled every ten minutes during the ingestion period. At the end of this period, a pre-exercise venous blood sample was again obtained immediately prior to the onset of exercise. The participants then commenced on a 60-minute self-paced (SP) cycling bout (Wattbike, Wattbike Ltd, Nottingham, UK). Although self-paced, the participants were encouraged to cover as much ground as possible in the 60-minute period (with a monetary incentive for the participant who covered the greatest cumulative distance over the four trials). The self-paced protocol was administered to provide ecological validity to the blood glucose and insulin responses during exercise, attempting to reflect the average energy expenditure during a moderate to difficult workout [5]. All participants were blinded to the distance covered, but given verbal cues as to the time completed. Average power (W) during the 60-minute ride and total distance covered (km) were recorded to assess performance efforts between trials.

At 15-minute intervals throughout the trial, subjects were required to consume 4 ml·kg^-1^BW of their prescribed drink over a 5-minute period (total carbohydrate (CHO) consumed during the trial conditions including CHO was 104.4 ± 11.3 g). Metabolic data was continuously measured and averaged in ten-minute intervals during exercise, with the exception of the drink intervals and venous blood draws, to provide an estimation of the respiratory exchange ratio (RER) via open circuit spirometry (OxyCon Pro, Jaegger, Hoechberg, Germany). Capillary samples were obtained during the venous sampling periods, while heart rate (HR) and rate of perceived exertion (RPE; [6]) were measured at 15, 30, 45 and 60 minutes. Venous blood was also sampled at 30 minutes and immediately following termination of the ride (60 minutes).

### Statistical analysis

All data are presented as mean ± SD. All data was assessed for normal distribution, homogeneity of variance, and independence of errors. Blood glucose and insulin was analyzed during resting conditions using a two-way (condition x time) repeated measures (RM) ANOVA design. Additionally, area under the curve (AUC) was calculated for blood glucose during the resting condition. The RM ANOVA was again employed on all data collected during the exercise period (blood, metabolic, cardiovascular and subjective data). All performance data was assessed using a one-way repeated measures ANOVA. Statistical analysis was done using Statistica Software (Tulsa, OK) and GraphPad Prism 3.0 (San Diego, CA). Post-hoc analysis was conducted for all significant interactions using Tukey’s HSD (*p* < 0.05).

### Results

#### Pre-exercise

**Figure 1:**
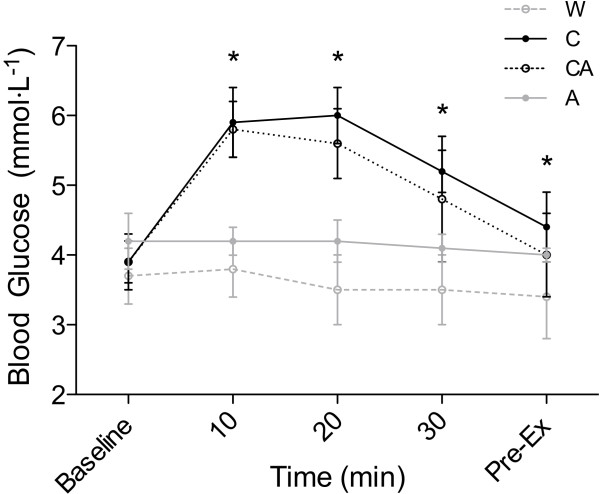
**Presented are the m ± SD profile of blood glucose during resting conditions (baseline, 10, 20, 30 minutes and pre-exercise (Pre-Ex)) after ingestion of either: 2% maltodextrin and 5% sucrose (C); 0.04% aspartame with 2% maltodextrin and 5% sucrose (CA); water (W); or 0.04% aspartame with 2% maltodextrin (A).** *Indicates C and CA significantly different from W and A (p < 0.05).

There was a significant interaction effect for blood glucose (p < 0.001), where both the C (5.7 ± 0.7 mmol·L^-1^) and CA (5.7 ± 0.4 mmol·L^-1^) trials resulted in higher resting BG values after 10 min post ingestion compared to W (3.9 ± 0.4 mmol·L^-1^) and A (4.2 ± 0.2 mmol·L^-1^) conditions (Figure 1). These differences, however, had subsided by 45 min, where there were no differences between conditions (C: 4.6 ± 0.6 mmol·L^-1^; CA: 4.2 ± 0.7 mmol·L^-1^; W: 3.5 ± 0.5 mmol·L^-1^; A: 4.0 ± 0.1 mmol·L^-1^). Although no difference between C and CA was evident in the mixed model design, the area under the curve (AUC) for C and CA was 213 and 202, respectively, indicating a lower blood glucose throughout the 45 min ingestion period in the CA condition compared to C. Similar differences were apparent between W and A, where A resulted in elevated BG values and AUC differences of 166 vs. 143. Serum insulin levels were also different at 45 min post ingestion between conditions (p = 0.005), where again the C and CA trials were significantly elevated compared to the W and A conditions (C: 16.2 ± 2.1 μlU·ml^-1^, CA: 16.2 ± 4.0 μlU·ml^-1^, W: 9.2 ± 1.3 μlU·ml^-1^, A: 8.9 ± 1.4 μlU·ml^-1^).

#### Exercise

There was no significant difference between trials for average power (p > 0.375; C: 190 ± 20 W, CA: 189 ± 20 W, W: 188 ± 17 W, A: 185 ± 20 W) or total distance covered (p > 0.152; C: 36.0 ± 1.2 km, CA: 35.8 ± 1.2 km, W: 35.9 ± 1.0 km, A: 35.5 ± 1.1 km), indicating a comparable amount of work was completed during each trial. Additionally, no metabolic (RER) (p > 0.840; C: 1.02 ± 0.04, CA: 1.03 ± 0.05, W: 1.03 ± 0.04, A: 1.02 ± 0.05), cardiovascular (HR) (p > 0.248; C: 167 ± 11 bpm, CA: 166 ± 15 bpm, W: 163 ± 15 bpm, A: 164 ± 9 bpm) or subjective measures (RPE) (p > 0.350; C: 15 ± 1, CA: 15 ± 1, W: 15 ± 1, A: 15 ± 1) were different between trials.

**Figure 2:**
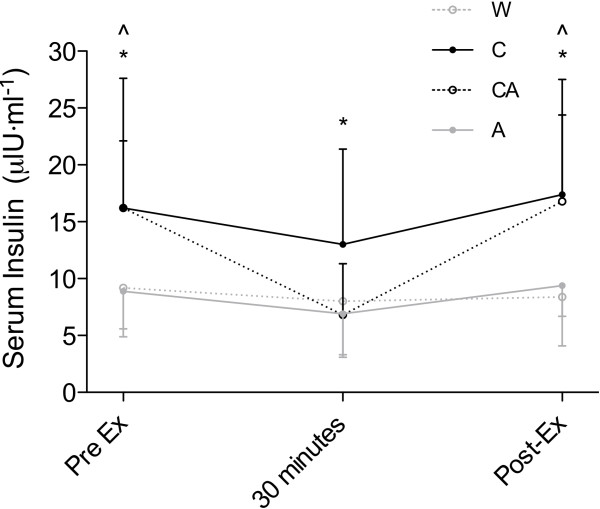
**Presented are the m ± SD profile of serum insulin concentrations during exercise (pre-exercise (Pre-Ex), 30 minutes and post-exercise (Post-Ex)) after ingestion of either: 2% maltodextrin and 5% sucrose (C); 0.04% aspartame with 2% maltodextrin and 5% sucrose (CA); water (W); or 0.04% aspartame with 2% maltodextrin (A).** *Indicates C significantly different from W and A (p < 0.05). ^Indicates and CA significantly different from W and A (p < 0.05).

There was no significant interaction for blood glucose during the 60 minutes of exercise (p > 0.824). However, there was a main effect for time (p < 0.015) and condition (p < 0.002) (Table 1). Similar to blood glucose, there was no interaction effect for serum insulin during the 60 minute ride (p > 0.079). However, there was a main effect for time (p < 0.002) and condition (p < 0.001) (Table 1; Figure 2).

**Table 1:** Presented are the m ± SD for pre-exercise (Pre-Ex), 30 minutes (30 min) and post-exercise (Post-Ex) blood glucose and serum insulin

	**Blood glucose (mmol·L**^**-1**^**)**			**Serum insulin (μlU·ml**^**-1**^**)**		
	**Pre-Ex**	**30 min**	**Post-Ex**	**Pre-Ex**	**30 min**	**Post-Ex**
C	4.6 ± 0.6	3.9 ± 0.7	4.4 ± 0.5	16.2 ± 5.9	13.0 ± 7.7	17.4 ± 7.0
CA	4.2 ± 0.7	3.8 ± 0.4	4.3 ± 0.9	16.2 ± 11.4	6.8 ± 4.5	16.8 ± 10.7
W	3.5 ± 0.5	4.1 ± 1.1	3.3 ± 0.7	9.2 ± 3.6	8.0 ± 4.9	8.4 ± 4.3
A	4.0 ± 0.1	4.2 ± 0.5	3.8 ± 0.7	8.9 ± 4.0	6.9 ± 3.6	9.4 ± 2.5

### Conclusions

The novel finding of this study was that despite a normal insulin response during the ingestion period (at rest), the combination of aspartame and carbohydrate (CA) led to significantly lower serum insulin levels during exercise than when compared to carbohydrate alone (C) (Figure 2). This decline during exercise, however, did not appear to influence blood glucose responses, as they were not different between the CA or C conditions (Table 1). This suggests that the reduction in insulin levels associated with aspartame ingestion observed in the current study may only be seen at a threshold of carbohydrate intake.

Although the results of the current study do not provide evidence for an underlying mechanism responsible for the variation in the exercise-induced insulin response, the disparity between insulin levels warrant further investigation with a larger cohort of clinically relevant subject populations (e.g. metabolic syndrome, diabetes, etc.). Additionally, we believe that these results may also need to be considered when designing nutrition-based, exercise intervention studies.

## Competing interests

The author(s) declare that they have no competing interests.

## Author’s contributions

JS was the principle investigator of the study. JS, RV, SA and DM conceived the study and participated in its design. RV and JS were responsible for the biochemical measurement and analysis. KH, JB, DP and CT aided with data collection and analysis. All authors read and approved the final manuscript.
